# Quality Assessment of a Foot-Mounted Inertial Measurement Unit System to Measure On-Field Spatiotemporal Acceleration Metrics

**DOI:** 10.3390/s26010246

**Published:** 2025-12-31

**Authors:** Marco Dasso, Grant Duthie, Sam Robertson, Jade Haycraft

**Affiliations:** 1 Institute for Health and Sport (IHeS), Victoria University, Melbourne, VIC 3000, Australia; marco.dasso@live.vu.edu.au (M.D.);; 2 High Performance Department, Western Bulldogs Football Club, Melbourne, VIC 3000, Australia; 3 School of Exercise Science, Australian Catholic University, Strathfield, NSW 2135, Australia; grant.duthie@acu.edu.au; 4 Sports Performance, Recovery, Injury, and New Technologies (SPRINT) Research Centre, Australian Catholic University, Strathfield, NSW 2135, Australia

**Keywords:** biomechanical technology, inertial measurement units, quality, validity, accuracy, running biomechanics

## Abstract

(1) Background: The use of wearable technology for assessing running biomechanics in field-based sports has increased in recent years. Inertial measurement units (IMUs) are low-cost, non-invasive devices capable of estimating spatiotemporal gait-related metrics during overground locomotion. This study evaluated the accuracy and concurrent validity of a foot-mounted IMU system for estimating sprinting kinematics. (2) Method: Twenty-five elite and sub-elite athletes completed four maximal 10-metre fly efforts, with their kinematics measured concurrently using a three-dimensional motion analysis system and IMUs. (3) Result: The foot-mounted IMU system’s root mean square errors for stride length and duration were 0.22 m and 0.04 s, respectively. Mean biases (95% level of agreement) were −0.67 $m\;\cdot \;s^{-1}$ (−1.19; −0.14) for peak velocity, −0.51 $m\;\cdot \;s^{-1}$ (−1.10; 0.09) for instantaneous velocity, and 0.17 $m\;\cdot \;s^{-2}$ (−1.04; 1.37) for instantaneous acceleration. Stride length, duration, and cadence were −0.07 m (−0.36; 0.23), 0.02 s (−0.02; 0.06), and −4.64 strides $\cdot \;\min^{-1}$ (−15.82; 6.53), respectively. (4) Conclusions: End users implementing this technology in research and practice should interpret this study’s findings relative to their analytical objectives, logistical resources, and operational constraints. Therefore, its adoption should be guided by the specific performance metrics of interest and the extent to which the system’s capabilities align with the outcomes the end user aims to achieve.

## 1. Introduction

Wearable technology provides practical tools to monitor athletes’ training loads and physical demands to enhance performance, reduce injury risk, and support recovery processes [1,2]. In field-based sports, global positioning systems (GPSs) and local positioning systems (LPSs) are among the most adopted wearable technologies for quantifying external load parameters such as distance, velocity, acceleration, and deceleration [3]. These systems provide insights into athletes’ movement profiles and tactical behaviours during competition. However, they cannot directly measure spatiotemporal kinematics, particularly the characteristics underpinning acceleration mechanics during sprinting [4,5,6]. As such, accurate assessments of gait-related metrics in field-based sports continue to rely heavily on laboratory-based optical systems such as three-dimensional (3D) motion capture and two-dimensional (2D) video analysis, which remain impractical for widespread field use due to their high cost, operational complexity, limited portability, and the substantial time and effort required for data coding, processing, and extraction by end users [7,8].

Recent advancements in wearable technology have facilitated the development of low-cost, portable, and non-invasive devices capable of estimating spatiotemporal gait-related metrics during overground locomotion [2,9]. Among these, inertial measurement units (IMUs), incorporating micro-electromechanical sensors (i.e., triaxial accelerometer, gyroscope, and magnetometer), capture linear acceleration and angular velocity across the sagittal, frontal, and transverse planes of motion [1,9]. By detecting characteristic signal patterns and applying dedicated algorithms, IMUs can estimate gait parameters related to on-field acceleration and sprint performance, such as ground contact time (GCT), flight time, stride length, stride duration, and cadence [1,10]. Despite their growing use, IMU-based gait analysis has primarily focused on accelerometer-derived signals, often neglecting the potential contribution of gyroscope and magnetometer data [11,12]. IMUs are typically placed on the lower back or lower limbs where acceleration along one or more axes is used to estimate spatiotemporal metrics [11,12]. In this regard, a recent scoping review of 231 studies employing IMU-based systems for gait analysis found that 64% assessed purely accelerometer magnitude in the vertical, anteroposterior, or mediolateral directions, while only 12% reported on kinematic metrics, with centre of mass (COM) displacement being the most frequently examined. Furthermore, 49% of the studies were conducted in controlled indoor settings, predominantly on treadmills [12]. While treadmill-based assessments enable standardisation of running speed and environmental conditions to facilitate device evaluation, they may compromise the ecological validity [11,12]. Biomechanical differences between treadmill and overground running have been well documented. Specifically, treadmill use has been associated with reduced COM vertical displacement and diminished peak propulsive and braking forces, likely due to the assistance of the moving belt, as well as increased GCT and variability in joint kinematics throughout the gait cycle [13]. As such, these biomechanical differences may not fully reflect the natural running mechanics in real-world environments, where locomotor dynamics are self-selected and unconstrained. Notably, the cited scoping review excluded studies focused solely on spatiotemporal variables such as speed, cadence, or step length [12]. Thus, while the review highlights broader methodological trends in IMU-based gait research, its exclusion criteria may limit its relevance for understanding prior assessments of spatiotemporal parameters.

While IMU-based systems are increasingly used in gait analysis, the literature addressing their application in overground running biomechanics and, specifically, gait event detection in field-based sports athletes, remains limited [12]. This is largely because most commercially available IMUs were not originally designed to capture spatiotemporal gait events during discrete phases of high-speed running and sprinting. Rather, they have been primarily used to quantify broader movement measures, such as COM velocity, acceleration, and deceleration, often integrated in GPS units [3,14]. Additionally, the conventional placement of GPS units on the upper thoracic spine, between the scapulae, further limits the ability of embedded micro-electromechanical sensors to detect gait-specific features accurately [1]. To address this limitation, foot-mounted IMUs have emerged as a promising alternative due to their proximity to the point of ground contact. This positioning enables the direct capture of triaxial inertial data at foot strike, thereby improving the detection of variables such as GCT, stride duration, and cadence [1,14]. For example, Young et al. [15] demonstrated the application of a zero-crossing signal processing technique to IMU data acquired on an instrumented treadmill to detect foot strike location and GCT [15]. However, while the method proved to be effective at lower running speeds, its accuracy declined at speeds exceeding 14 $\mathrm{km}\;\cdot \;h^{-1}$, when compared to a 3D motion analysis system, thus highlighting limitations in high-speed gait event detection using IMUs [15]. Moreover, research evaluating the quality of foot-mounted IMUs has primarily focused on walking and slow-speed running gait or on populations presenting clinical pathologies [11,12,16,17].

The current regulatory landscape for sports technologies lacks formal statutory standards governing their development, and manufacturers are not obligated to demonstrate the quality of the data their systems produce. As a result, the level of methodological rigour applied during product development and validation varies considerably across technologies. Consequently, independent appraisal of measurement quality remains critical before implementing devices such as IMUs, GPSs, LPSs, and camera-based systems for the assessment of human motion [18,19]. Rigorous evaluations ensure confidence in the measurements provided to end users across various applications in both research and applied settings. In this context, accuracy and concurrent validity are among the most employed methods for assessing sports technology, as they determine the degree to which a device’s outputs correspond to those of a gold standard or previously validated system for similar measurements [19,20]. However, despite the increasing prevalence of wearable technology in the contemporary sports scenario, fewer than 10% of commercially available systems have undergone formal validation against accepted criterion methods [11], underscoring the need for evidence-based assessment.

One such device, PlayerMaker™ (PlayerMaker™, Tel Aviv, Israel), is a wearable IMU-based motion analysis system originally developed for performance monitoring in soccer. Mounted on the feet, the system is capable of estimating bilateral spatiotemporal gait parameters, including stride length, stride duration, and stride cadence, in addition to providing velocity and acceleration metrics relative to the COM [1,21]. Although PlayerMaker™ has been adopted across various sporting contexts, studies have yet to comprehensively evaluate its accuracy in estimating these metrics. Therefore, this study aims to assess the measurement quality of PlayerMaker™ within the context of running biomechanics, with emphasis placed on the acceleration-related characteristics relevant to high-speed running and sprinting. Specifically, guided by the Sport Technology Quality Framework [19], this investigation seeks to determine the accuracy and concurrent validity of PlayerMaker™ spatiotemporal gait-related metrics, in comparison to a criterion 3D motion analysis system, integrating infrared cameras and force plates measurements, in order to evaluate its suitability for field-based applications.

## 2. Materials and Methods

### 2.1. Participants

Twenty-five elite and sub-elite athletes (16 females and 9 males; 22.9 ± 5.34 years) took part in the study. The cohort comprised Australian footballers (*n* = 19), soccer players (*n* = 4), and track and field sprinters (*n* = 2). Participants’ training regimes ranged from three to five moderate-to-high-intensity training sessions per week, incorporating both on-field running and gym-based strength training. The study received ethical approval from the university’s Human Research Ethics Committee (HRE24-044), and written informed consent was obtained from all participants prior to data collection.

### 2.2. Procedure

**Figure 1:**
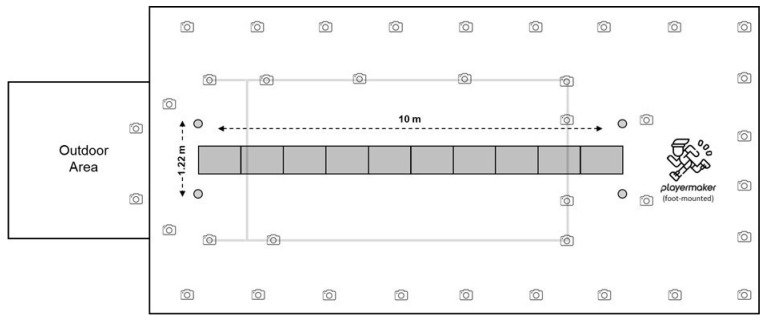
Biomechanics laboratory setup highlighting the 10 m fly capturing space and the 3D motion capture system.

Participants completed a standardised warm-up protocol consisting of mobility exercises, dynamic stretching, and running activation drills. This was followed by three familiarisation sprints performed at 70%, 80%, and 90% of their maximal velocity. After the warm-up, participants undertook four maximal linear sprint accelerations (10 m fly), with two to three minutes of passive recovery between repetitions. Each sprint commenced from a 10 m approach run before entering the 10 m fly zone, which was positioned over an instrumented runway of force plates, and concluded with a 10 m deceleration to a complete stop (Figure 1).

During data collection, participants wore a custom Lycra and Velcro VICON motion capture lower-body suit fitted with 18 retro-reflective spherical markers (14 mm diameter) and four rigid-body clusters, each containing four markers, positioned on key anatomical landmarks (i.e., hips, upper leg, lower leg, and feet). Marker placement followed the VICON Plug-In Gait (PiG) lower-body model for three-dimensional motion analysis [22], with additional rigid-body clusters on the thighs and shanks of participants. The PiG VICON model was selected, as it most closely aligns with the experimental technology’s method of deriving velocity and acceleration relative to the COM, thereby ensuring consistency in motion analysis. Three-dimensional spatiotemporal metrics were collected using a 38-infrared camera 3D motion capture system (12 × MX T40-S, 9 × Vantage 5, 15 × Vantage 8, 2 × Vantage 16; Vicon Motion System Ltd., Oxford, UK) operating at 100 Hz. Additionally, 10 ground-embedded force plates (AMTI BMS600900; AMTI, Watertown, MA, USA) sampling at 1000 Hz (Figure 1) were used to determine GCT and ground reaction force. Cameras and force plates were synchronised via VICON Nexus software (version 2.15; Vicon Motion System Ltd., Oxford, UK), through which all trials were recorded. The capturing area was demarcated using one set of cones at 0 m (start) and 10 m (finish) mark, with the two cones in each set placed 1.22 m apart (Figure 1). Furthermore, four VICON reflective spherical markers (25 mm diameter) were positioned adjacent to each start and finish cone, enabling precise identification of the 10 m fly data collection area within the 3D motion analysis environment.

**Figure 2:**
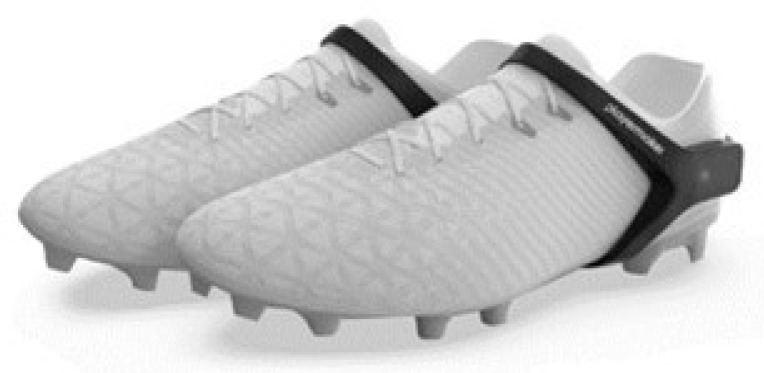
PlayerMaker™ system fitted to the later-posterior aspect of the foot [24].

Spatiotemporal gait-related metrics were simultaneously collected using the PlayerMaker™ system, which comprises an IMU housed within a customised silicone strap and securely placed onto the lateral-posterior aspect of each participant’s running shoe (Figure 2). The PlayerMaker™ IMU incorporates a triaxial accelerometer (±16 g), triaxial gyroscope ($2000{}^{\circ}\cdot \;s^{-1}$), and triaxial magnetometer (±1200 μT) from the MPU-9150 tracking module (InvenSense, San Jose, CA, USA), sampling at 1000 Hz, which derives whole-body velocity-based metrics using data generated by the built-in micro-electromechanical sensors, using a combination of proprietary gait-tracking and foot-based event detection algorithms [1,23]. Data collection was managed via the PlayerMaker™ application (version 3.58.1; PlayerMaker™; Tel Aviv, Israel) installed on an iPad (9th generation; iOS 17.6.1; Apple Inc., Cupertino, CA, USA). The application communicated with the IMUs through Bluetooth technology to initiate and synchronise recordings. Additionally, to track the position of the PlayerMaker™ IMUs within the 3D motion capture space, a retro-reflective spherical marker (14 mm diameter) was securely attached to each IMU of both feet.

### 2.3. Data Processing

Prior to data extraction from VICON Nexus, each trial was reconstructed and gap-filled using the Woltring spline algorithm over maximum gaps of five frames. Subsequently, all trials were manually labelled. Foot strike and toe-off events were identified from vertical ground reaction force traces (1000 Hz) recorded by the force plates and used to infer gait-related variables of interest according to the definitions provided by PlayerMaker™ (PlayerMaker™, email, 11 April 2025). A threshold of 50 N in vertical ground reaction force was used to determine the onset of foot contact on the force plates as per best-practice recommendations [25,26]. Specifically, stride length was defined as the cumulative displacement between two successive steps from the same foot (i.e., left to left or right to right), calculated using the reflective markers affixed to the PlayerMaker™ devices as spatial reference. Stride duration was determined as the interval between the foot strike events of the same foot during two consecutive steps. Stride cadence was computed as the number of strides per minute. Additionally, events were created to establish the entry and exit of participants from the 10 m fly area. These events were determined using the frame corresponding to when the participant crossed the capturing space (0 m) with the anterior foot toe marker and when they exited the space (10 m) with the posterior foot toe marker.

Before data extraction, PlayerMaker™ data were edited using the system’s dashboard (version 3.45.1.2; PlayerMaker™; Tel Aviv, Israel) for computer, during which trials were defined and segmented accordingly. PlayerMaker™ and VICON Nexus data (i.e., trajectories and force plates-based events) were then downloaded and imported into customised Microsoft Excel™ (version 2301, Microsoft Corporation, Santa Rosa, CA, USA) spreadsheets, with processing and synching executed using RStudio^®^ statistical computing software (version 2024.09.1; RStudio, Boston, MA, USA). VICON trial exports were cut to include only data collected within the capturing space and wrangled using readr, zoo, data.table, tidyr, dplyr, and signal R packages [27,28,29,30,31,32].

Prior to data synchronisation, VICON COM linear velocity components from the x, y, and z axes (automatically computed by VICON Nexus via the World Root joint function) were converted into a resultant value to account for force applications and COM displacement occurring horizontally, vertically, and laterally as participants moved in a forward linear direction. [33,34]. The resultant COM acceleration was subsequently derived from the resultant velocity by numerically differentiating the velocity signal using a central difference method and reported as $m\;\cdot \;s^{-2}$. Consequently, 4th-order low-pass Butterworth filters with frequencies of 7.32 Hz and 6.03 Hz were applied to velocity and acceleration datasets, respectively, to reduce noise and smooth signal variation. These frequencies were determined through residual analyses (cut-off frequency range, 4 Hz; 20 Hz) using data.table and caTools R packages (version 2024.09.1; RStudio, Boston, MA, USA) [29,35]. Although PlayerMaker™ collects instantaneous velocity and acceleration at 1000 Hz, these variables are provided to end users at 10 Hz following proprietary algorithmic processing. In contrast, stride length, stride duration, and stride cadence are reported as discrete gait events based on when the event occurred rather than at a fixed sampling frequency. As this study aimed to assess PlayerMaker™ variables provided to the end user, no backend data were analysed.

Data synchronisation for instantaneous velocity and acceleration between PlayerMaker™ and VICON occurred using the reference time from the VICON analysis system corresponding to when participants were within the 10 m capture zone. PlayerMaker™ data were upsampled from 10 Hz to 100 Hz to match the sampling frequency of the VICON system. Successively, datasets from both technologies were aligned and shifted to the smallest root mean square error (RMSE) using instantaneous velocity as reference [36]. This process was conducted using shiny, ggplot2, plotly, data.table, and tidyverse R packages [29,37,38,39,40]. Stride length, stride duration, and stride cadence were manually synchronised by matching each PlayerMaker™ stride-related event to the corresponding event identified in VICON Nexus, as PlayerMaker™ reports these variables only at the moment each event occurs and not at a uniform sampling frequency.

### 2.4. Statistical Analyses

**Figure 3:**
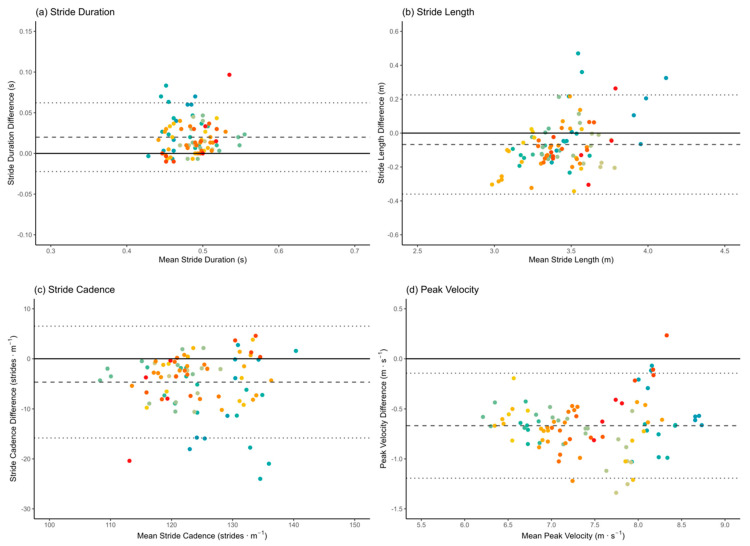
Bland–Altman plots comparing PlayerMaker™ and VICON 3D motion capture system for spatiotemporal gait-related metrics reporting the mean difference and 95% LoA for all trials (*n* = 92). **Note**: (**a**) stride duration, (**b**) stride length, (**c**) step cadence, (**d**) peak velocity. Dashed line = mean difference, dotted lines = 95% LoA (±1.96 × standard deviation of differences). Each colour represents a participant.

A total of 92 trials were used for the analysis. Eight trials were discarded due to technical issues with processing PlayerMaker™ data from two participants. Therefore, stride duration, stride length, and stride cadence were computed on 265 strides (*n* = 132 left, *n* = 133 right). Differences in left and right stride counts occurred because participants ran freely through the 10 m capture zone without constraints on which foot initiated or ended the sequence. This approach preserved ecological validity, thereby yielding natural between-individual variation in captured stride counts (Table 1). Peak velocity, instantaneous velocity, and instantaneous acceleration were analysed for each trial using the synchronised, aligned data. The distribution of all variables of interest was evaluated using the Kolmogorov–Smirnov test, which revealed that stride cadence collected by VICON, as well as stride duration and instantaneous velocity and acceleration from both PlayerMaker™ and VICON, were non-normally distributed. Therefore, non-parametric statistical analyses were employed for all variables. Descriptive analyses, mean absolute error (MAE), RMSE, and mean bias, along with the 95% level of agreement (LoA), were employed to assess the average magnitude of errors between predicted and actual values, as well as any systematic overestimation or underestimation in the PlayerMaker™ system [19,20]. For the calculation of the LoA, only the first trial from each participant was used to avoid the dependency associated with repeated measures from the same individual. For comparative purposes, LoA values were also calculated using the pooled dataset, which incorporated all trials across participants (Table 2). Spearman rank-order correlation coefficients (ρ) were computed to evaluate the linear relationship between technologies’ outputs [41,42]. The criteria for determining the correlation magnitude were established as follows: trivial, 0 to <0.1; small, 0.1 to <0.3; moderate, 0.3 to <0.5; large, 0.5 to <0.7; very large, 0.7 to <0.9; nearly perfect, 0.9 to 1.0 [43]. Additionally, Bland–Altman plots with 95% LoA, including all trials, were produced to visually represent the agreement and distribution of differences both between and within participants across the two systems (Figure 3). Prior to this, dataset heteroscedasticity was assessed using Spearman correlations between the absolute Bland–Altman differences and their corresponding means, and no evidence of heteroscedasticity was found for any discrete measure. Log-transformed Bland–Altman analysis was also applied to stride-related metrics and to peak and instantaneous velocity to account for proportional error and to express agreement on a relative scale (i.e., normalised to the magnitude of the measurement). Conversely, instantaneous acceleration was excluded from this analysis because it contains negative and near-zero values that violate the assumptions of the log–ratio method [44]. Statistical analyses and plots were computed using readr, purrr, data.table, ggplot2, irrICC, lmtest, and tidyverse R packages [27,29,37,40,45,46].

## 3. Results

Table 2 presents the statistical outcomes comparing spatiotemporal gait-related metrics derived from PlayerMaker™ and VICON 3D motion capture systems. Spearman’s rank-order correlation coefficients indicated nearly perfect correlations for peak and instantaneous velocity and a very large correlation for stride length. Large correlations were observed for stride duration and cadence. In contrast, instantaneous acceleration exhibited a trivial correlation, suggesting limited agreement between the two technologies for this variable. Mean bias analyses revealed that PlayerMaker™ consistently underestimated stride length and cadence, and both peak and instantaneous velocity, when compared with VICON. Conversely, PlayerMaker™ overestimated stride duration and instantaneous acceleration. Furthermore, stride-related metrics were associated with relatively small MAE and RMSE values, indicating acceptable levels of measurement accuracy. In contrast, instantaneous acceleration demonstrated larger MAE and RMSE values, reflecting greater variability and reduced agreement between PlayerMaker™ and the criterion system for this variable (Table 2).

**Table 2:** Accuracy and concurrent validity results.

Variable	Technology	Mean ± SD	MAE	RMSE	Mean Bias (95% LoA)	Spearman’s Correlation (ρ)
Stride duration (s)	Playermaker	0.43 ± 0.03	0.02	0.04	0.02 (−0.02; 0.06)	0.60
	VICON	0.47 ± 0.02				
Stride length (m)	Playermaker	3.37 ± 0.29	0.17	0.22	−0.07 (−0.36; 0.23)	0.72
	VICON	3.45 ± 0.19				
Stride cadence (strides $\cdot \;\min^{-1}$)	Playermaker	122.08 ± 9.05	6.25	8.94	−4.64 (−15.81; 6.53)	0.61
	VICON	126.47 ± 7.57				
Peak velocity ($m\;\cdot \;s^{-1}$)	Playermaker	7.10 ± 0.68	0.67	0.72	−0.67 (−1.19; −0.14)	0.92
	VICON	7.76 ± 0.66				
Inst. velocity ($m\;\cdot \;s^{-1})$	Playermaker	6.80 ± 0.60	0.52	0.56	−0.50 (−1.10; 0.09)	0.91
	VICON	7.30 ± 0.63				
Inst. acceleration ($m\;\cdot \;s^{-2}$)	Playermaker	0.19 ± 0.61	0.49	0.64	0.17 (−1.04; 1.37)	0.19
	VICON	0.02 ± 0.21				

**Abbreviations:** SD = standard deviation, MAE = mean absolute error, RMSE = root mean square error, LoA = level of agreement, Inst. = instantaneous.

For each variable, the 95% LoA is reported for the first trial of each participant (*n* = 23), with secondary values presented in parentheses representing results derived from the pooled dataset (*n* = 92), which included multiple trials per participant (Figure 3). Stride-related metrics exhibited similar LoA values between the single-trial and pooled-trial approaches. Specifically, for stride length, stride duration, and stride cadence, LoA were −0.32; −0.19 m (−0.36; −0.23), −0.02; 0.07 s (−0.02: 0.06), and −16.16; 5.94 strides $\cdot \;\min^{-1}$ (−15.82; 6.53), respectively. For peak and instantaneous velocity and for instantaneous acceleration, LoA ranges were −1.19; −0.21 $m\;\cdot \;s^{-1}$ (−1.19; −0.14), −1.07; 0.03 $m\;\cdot \;s^{-1}$ (−1.10; 0.09), and −1.11; 1.42 $m\;\cdot \;s^{-2}$ (−1.04; 1.37). Additionally, log-transformed Bland–Altman analysis performed on the pooled dataset expressed agreement on a relative scale. Stride duration showed a proportional bias and LoA of 3.90% (−8.02; 17.39), stride length of −2.31% (−13.00; 9.69), and stride cadence of −3.56% (−14.67; 8.99). Peak and instantaneous velocity were −8.68% (−14.88; −2.03) and −6.92% (−14.16; 0.92), respectively.

## 4. Discussion

This study aimed to evaluate the accuracy and concurrent validity of spatiotemporal gait-related metrics, obtained from PlayerMaker™ during sprinting, against a criterion 3D motion analysis system. Overall, the results demonstrated variable levels of agreement between the two systems. Stride duration, stride length, and stride cadence exhibited small proportional mean biases and wide proportional LoA ranges. However, when results were assessed on an absolute scale, stride duration and stride length demonstrated small mean biases, narrow LoA, and low error magnitudes, indicating that PlayerMaker™ and the VICON system produce comparable outputs. Although some variability in rank-order correlations was observed across these metrics, the findings support the use of PlayerMaker™ to capture stride duration and stride length during sprinting. In contrast, stride cadence showed larger error magnitudes and a wider LoA, reflecting increased variability and reduced measurement accuracy. Peak and instantaneous velocity showed excellent correlation with VICON, suggesting that the system reliably tracked the temporal pattern of velocity changes. However, both metrics demonstrated systemic underestimation, accompanied by moderate error magnitudes and LoA ranges that indicated some degree of measurement variability. This pattern of variability and consistent systemic underestimation was also evident when proportional agreement was assessed using log-transformed Bland–Altman analysis. Collectively, these findings suggest that although PlayerMaker™ may underestimate absolute velocity values, it maintains a consistent representation of velocity trends across trials. Conversely, instantaneous acceleration showed reduced agreement between systems, with wide limits of agreement, low correlation, and substantial variability, indicating limited ability to capture rapid acceleration fluctuations during high-speed movement. It should be acknowledged that while proportional analyses are critical for statistical interpretation and cross-metric comparison, absolute measures are more informative for evaluating practical relevance and guiding monitoring and decision-making in applied sports performance settings [47].

The findings related to velocity metrics in this study align with those reported in previous investigations using PlayerMaker™, which have shown instantaneous velocity at speeds exceeding 6 $m\;\cdot \;s^{-1}$ during linear sprint tasks to be underestimated (mean ± SD: −0.54 ± 0.34 $m\;\cdot \;s^{-1}$; 95% LoA: −1.22; 0.12  $m\;\cdot \;s^{-1}$) compared to criterion systems, although agreement between technologies improved during lower-velocity drills involving changes of direction [21]. This indicates that both movement speed and task type may influence PlayerMaker™ measurement accuracy [21]. While the present study did not assess tasks involving directional changes or different velocity thresholds, Bland–Altman analysis revealed greater variability in peak velocity among participants achieving higher velocities, supporting the notion of reduced measurement agreement at elevated speeds. Such velocity-dependent patterns likely reflect the combined influence of mechanical and computational challenges that IMU-based systems encounter when capturing rapid changes in limb or segment kinematics. At higher speeds, the magnitude and frequency of acceleration (and deceleration) increase, amplifying sensor noise and introducing small timing errors in detecting gait events [15,48]. The PlayerMaker™ system estimates velocity through a gait-tracking algorithm that identifies limb orientation and translation across the gait cycle, detecting events such as heel strike, toe-off, zero-velocity, zero-height, and non-gait patterns, with raw accelerometer and gyroscope signals processed using a Kalman filter to estimate orientation, velocity, and position vectors [1,21]. However, as running velocity increases, the stance phase becomes markedly shorter, and kinematic transitions (e.g., from heel strike to toe-off) become more abrupt, reducing the time available for accurate zero-velocity updates. This constrains the filter’s ability to correct for drift, increasing the potential for cumulative integration error in the reconstructed velocity signal [15,49]. As such, these factors provide a plausible explanation for the reduced agreement observed during faster sprinting efforts. Similar underestimations were found between PlayerMaker™ and the criterion systems for instantaneous acceleration in this study; however, unlike velocity, acceleration is directly captured by the IMU’s accelerometer. This highly sensitive component detects changes in capacitance as a proof mass shifts between fixed electrodes in response to motion [50,51]. This direct measurement is particularly vulnerable to high-frequency fluctuations generated by sensor mount vibration and impact shock during foot–ground contact in sprinting. Such artefacts can obscure the true underlying signal, especially during rapid force application phases, thereby diminishing agreement with the criterion system [15].

No prior research has specifically assessed PlayerMaker™ for measuring stride length, stride duration, or stride cadence. Nonetheless, studies using other foot-mounted IMU devices provide useful, albeit context-dependent, benchmarks for interpreting the present findings. Consistent with the small underestimation observed in this study, evidence from linear sprints has shown mean biases of −0.75% (LoA = ±6.39%) and −2.51% (LoA = ±8.54%) at 80% and 100%, of maximal effort, respectively, when a foot-mounted IMU was compared with a video-based system [52]. Similarly, under walking conditions, a mean difference of 0.03 m was reported when comparing a foot-mounted IMU with a 3D motion capture system [53]. Collectively, these findings suggest that stride length estimates are relatively unaffected by movement velocity, likely because each stride is processed as a discrete cycle anchored to robust gait events, with integration drift effectively constrained through zero-velocity updates [49]. The agreement between PlayerMaker™ and VICON for stride duration and stride length observed in the current study aligns with stride-related findings reported in previous investigations, supporting the suitability of foot-mounted IMUs for on-field assessment across different phases of running technique [54,55]. In contrast, the reduced agreement observed for stride cadence reflects a well-recognised limitation of IMU-based gait analysis. Because cadence is derived as a ratio of stride events, even small timing errors in detecting heel strike or toe-off events are amplified in the final calculation. Stride duration is comparatively less sensitive to timing inaccuracies, as these errors represent a small proportion of the full stride interval, whereas cadence, calculated as the inverse of stride duration, magnifies these discrepancies. Furthermore, this issue becomes more pronounced at higher running velocities, where shortened stance and swing phases increase the proportional impact of event-detection inaccuracies on cadence estimates [56,57]. Overall, this suggests that foot-mounted IMUs are well-suited for on-field assessment of stride length and duration. However, caution is warranted when interpreting cadence measures, especially at faster speeds.

Evidence on stride duration from foot-mounted IMUs during sprinting is scarce, with most existing research focusing on spine-mounted sensors and evaluations conducted during walking or sub-maximal running [11]. Notably, one study reporting on effort at maximal sprinting velocity demonstrated excellent agreement with criterion measures, with mean differences and errors of 0.01 s when lumbar-mounted IMU estimates were compared against force plates and high-speed video [58]. While these results are congruent with the agreement observed between PlayerMaker™ and VICON in the present study, the literature reports that sensor placement appears to influence signal fidelity, likely due to the closer proximity of foot-mounted devices to the point of foot–ground contact [56]. This effect becomes even more apparent when stride duration is derived from GPS-integrated accelerometers mounted on the thoracic region, with evidence reporting a weak association with force plate measurements, highlighting the limitations of distant sensor placements for precise gait event detection [59]. Furthermore, unlike IMUs, accelerometers cannot capture angular velocity, which may limit their ability to robustly identify key gait events such as heel strike and toe-off. The absence of rotational data can exacerbate the poor agreement observed with criterion systems and reinforces the value of foot-mounted IMUs for more accurate assessment of stride duration in high-speed running and sprinting [56,58]. As such, sensor placement and signal modality influence measurement fidelity, with foot-mounted IMUs showing greater potential than torso-mounted accelerometers for assessing stride duration during sprinting.

This study presented several limitations. A key constraint was the restricted testing space, which limited trials to 10 m. Although this distance was sufficient to capture fundamental kinematic features of sprint performance, it constrained the collection of a larger, heterogeneous dataset needed for a more comprehensive analysis of movement dynamics. Particularly, the number of strides obtained per participant may have increased stride-to-stride variability, influenced the magnitude of statistical error estimates, and contributed to wider LoA. A limitation arose from deriving VICON acceleration from velocity data using numerical differentiation, a process that may have attenuated transient acceleration peaks of short duration. Conversely, direct accelerometer-based measurements, such as those used by PlayerMaker™, may retain rapid fluctuations with higher temporal fidelity but are more susceptible to high-frequency artefacts. These differences in signal characteristics may have influenced the comparison between technologies [60]. Beyond these inherent signal differences, methodological divergences between systems, including the use of distinct filtering approaches (Kalman vs. Butterworth) and sampling frequencies, may also have influenced the comparison. A further challenge in this study was aligning stride-related metrics between systems, as the PlayerMaker™ outputs were event-based rather than reported at a defined sampling frequency (e.g., 10 Hz vs. 100 Hz vs. 1000 Hz). This reporting format did not directly align with the criterion system’s time-series-based measurements, necessitating manual synchronisation. Although instantaneous velocity and acceleration trace synchronisation was used to minimise misalignment of stride-related metrics, the nature of PlayerMaker™’s event-based reporting means that some degree of temporal mismatch and consequent influence on error estimates remain possible. Future studies should aim to address these issues by employing longer running distances and analysing PlayerMaker™ backend data to facilitate a homogenous methodology for deriving acceleration and to improve the precision of stride-related metric synchronisation with the criterion system.

## 5. Conclusions

The integration of non-invasive, low-cost wearable technologies such as IMU-based systems requires careful consideration of accuracy requirements in applied biomechanics assessments. In sport performance contexts, a high degree of measurement precision and reliability is often desirable, as gait phases at high velocities occur over short durations, and even subtle timing differences in gait event detection can influence the evaluation of neuromuscular function. Although there is no established consensus on the minimum acceptable level of agreement for IMUs in estimating spatiotemporal gait-related metrics, the tolerable magnitude of error likely depends on factors such as the testing environment (e.g., laboratory versus field conditions) and the characteristics of the movement task (e.g., high speed versus submaximal efforts). Playermaker™ offers practical value for assessing and monitoring running biomechanics in real-world sport environments, providing ecologically valid insights that may not be attainable with laboratory-based systems. Its suitability should therefore be considered in relation to the specific performance objectives, context of application, and precision required to inform training and monitoring practices.

## Figures and Tables

**Table 1:** Participants’ trials and strides classification.

Participants	Sport	Trials (*n*)	Left Strides (*n*)	Right Strides (*n*)
1	VFLW	4	8	8
2	VFLW	4	8	4
3	VFLW	4	8	4
4	VFL	4	4	7
5	VFLW	4	4	4
6	AFLW	4	4	8
7	VFLW	4	4	8
8	VFLW	4	5	7
9	AFLW	4	8	4
10	AFL	4	4	4
11	VFL	4	8	4
12	VFL	4	4	6
13	VFLW	4	4	8
14	VFLW	4	4	7
15	VFLW	4	8	4
16	Track and field	4	7	4
17	AFLW	4	8	4
18	AFLW	4	4	8
19	VFLW	4	8	4
20	A-League Academy	4	4	8
21	A-League Academy	4	4	8
22	A-League Academy	4	8	4
23	A-League Academy	4	4	6
Total		92	132	133

**Abbreviations:** AFL = Australian Football League, AFLW = Australian Football League Women, VFL = Victorian Football League, VFLW = Victorian Football League Women, A-League = Australian soccer professional league.

## Data Availability

The original contributions presented in this study are included in this article. Further inquiries can be directed to the corresponding author.
